# Pulmonary Recruitment Maneuver Reduces Shoulder Pain and Nausea After Laparoscopic Cholecystectomy: A Randomized Controlled Trial

**DOI:** 10.1007/s00268-021-06262-6

**Published:** 2021-09-05

**Authors:** E. Kihlstedt Pasquier, E. Andersson

**Affiliations:** 1 Department of Surgery, Vrinnevi Hospital, Gamla Övägen 25, 603 79 Norrköping, Sweden; 2 Department of Surgery and Department of Clinical and Experimental Medicine, Linköping University, Norrköping, Sweden

## Abstract

**Background:**

Pain and nausea are common after laparoscopic surgery. This prospective, randomized, controlled trial aimed to investigate postoperative pain and as a secondary endpoint nausea, when performing a ventilator-piloted Pulmonary Recruitment Maneuvre (PRM) at the end of laparoscopic cholecystectomy.

**Method:**

Patients having elective laparoscopic cholecystectomy were randomized to either ordinary exsufflation or ventilator-piloted PRM, to evacuate intra-abdominal carbon dioxide (CO_2_) before abdominal closure. A questionnaire with numeric rating scales (NRS) was utilized to evaluate pain and nausea at five occasions during 48 h following surgery. Analgesic and antiemetic treatment was also analyzed.

**Results:**

147 patients were analyzed, 76 receiving PRM and 71 controls. Overall pain was well controlled, with no significant difference between the groups regarding incidence (*P*=0.149) nor intensity (*P*=0.739). Incidence of shoulder pain was lower in the PRM group during the 48 postoperative hours, 44.7% versus 63.4% (*P*=0.023). The number needed to treat (NNT) to reduce shoulder pain was 6 (95% Confidence Interval, CI, 2.9–35.5) for the 48-h period. Incidence of nausea was lower in the PRM group during the 48-h period, 51.3% versus 70.4% (*P*=0.018). NNT was 6 (95% CI 2.9–27.4) for the 48-h period. Nausea intensity was lower in the PRM group during the 48 h (*P*=0.025). Fewer in the PRM population required antiemetics, 25.0% versus 42.3% (*P*=0.027).

**Conclusion:**

A ventilator-piloted PRM at the end of laparoscopic cholecystectomy reduced incidence of shoulder pain, and incidence and intensity of nausea. *Clinical trial registration* www.clinicaltrials.gov. Identifier: NCT03026543.

**Supplementary Information:**

The online version contains supplementary material available at 10.1007/s00268-021-06262-6.

## Introduction

Cholelithiasis affects up to 20% of the population in developed countries. The surgical treatment of choice worldwide is laparoscopic cholecystectomy [1]. Pain and nausea following the procedure are important reasons for prolonged hospital stay and readmissions [2]. In a previous study, we found that a pulmonary recruitment maneuvre (PRM) reduced pain after bariatric surgery [3], consistent with results from other studies, mainly gynecological [4–10]. This trial aimed to investigate postoperative pain when performing a ventilator-piloted PRM at the end of laparoscopic cholecystectomy. Nausea was analyzed as a secondary endpoint.

The theory behind the benefit of PRM is that part of the post-laparoscopic pain is due to trapped intra-abdominal CO_2_ [11–15]. The entrapped gas may irritate or stretch the diaphragm, cause local acidosis and supposedly initiates referred shoulder pain via its effect on the phrenic nerve. [4, 16] PRM lowers the diaphragm, which in turn raises the intra-abdominal pressure, hence mechanically facilitates evacuation of CO_2_ [4–6].

## Methods

This prospective, randomized, controlled trial was conducted in a secondary level hospital, engaging both experienced staff and staff under education, aspiring results of clinical significance. Participants, postoperative personnel and the investigator registering data were blinded to group affiliation. All study procedures were approved by the Regional Ethical Review Board in Linköping, Sweden (2014/120-31). The trial was registered at www.clinicaltrials.gov; registration number: NCT03026543.

Inclusion criteria: adults (>18 years); American Society of Anesthesiologists (ASA) physical status classification I-II (not including body mass index as a health variable); patients scheduled for elective laparoscopic cholecystectomy. Written consent was obtained. Exclusion criteria: acute cholecystitis, pancreatitis or cholangitis; conversion to open surgery; endoscopic retrograde cholangiopancreatography (ERCP) during or after surgery; extra surgical procedure; complications classified as Clavien-Dindo grade (CD)≥II [17].

Patients were consecutively enrolled and randomized to one of two equally sized groups; intervention with PRM or control. Block randomization was computer-generated by an independent statistician, and the allocation decision was placed in sealed, opaque, sequentially numbered envelopes. The envelope in turn followed the participant to theater, where it was opened by the anesthetic staff. Preoperatively, participants filled in a health declaration. Six weeks of non-smoking was a prerequisite for surgery.

Four-port laparoscopic cholecystectomy in anti-Trendelenburg position was performed. Gas pressure was maximum 12 mm Hg. General anesthesia was monitored by an anesthesiologist and nurse. Induction was achieved with either remifentanil and propofol or alfentanil and thiopental, the latter for patients with gastroesophageal reflux. Maintenance anesthesia was with remifentanil as analgesic, and propofol or sevoflurane in oxygen-enriched air as hypnotic. Pressure regulated volume control was standard ventilator mode. The surgeon was either an attending or a resident assisted by a senior colleague. Peroperative cholangiography was performed, according to Swedish routine. If a stone were obstructing the bile duct, an ERCP was performed during surgery or scheduled the following day, and the patient would then be excluded from the study.

In both groups, intra-abdominal CO_2_ was evacuated passively through the open sleeve valve of the epigastria port, while the surgeon applied gentle abdominal pressure. In the intervention group, a PRM was performed according to a specific protocol [3] before removal of the port, using the ventilator (GE Datex-Ohmeda Aisys, Madison, WI, United States). During one-minute of pressure-controlled ventilation, the patient received 6 breaths with a total pressure of 40 cm H_2_O. CO_2_ is heavier than air [18], wherefore PRM was performed with the patient in supine position to avoid retained CO_2_ in the abdominal cavity. After gas exsufflation, the subumbilical fascia was sutured. Incisions were sutured intracutaneously. Local anesthesia (20 ml bupivacaine hydrochloride 5 mg/ml with epinephrine 5 ug/ml) was infiltrated subcutaneously around trocar incisions.

Participants received oral paracetamol preoperatively. 30 mg ketorolac and/or morphine was administered intravenously (IV) near the end of surgery. The morphine dose was documented. Droperidol (0.5 mg IV) and betamethasone (4 mg IV) were given as antiemetic prophylaxis. Those with a history of postoperative nausea and vomiting (PONV) also received 4 mg ondansetron IV. The analgesic regimen postoperatively consisted of 1 g paracetamol four times daily. In the post-anesthesia care unit IV opioids were given as needed, while a 5 mg immediate-release oxycodone capsule was offered as rescue analgesia in the surgical ward. While hospitalized, patients experiencing nausea were treated with ondansetron IV. The doses administrated were recorded for follow-up.

Participants completed a 48-h postoperative questionnaire, with the same questions asked at five different occasions, and the replies given by ticking boxes. Intensity of pain and nausea, respectively, was evaluated with a numeric rating scale (NRS), ranging from 0 (no pain/nausea) to 10 (worst imaginable pain/nausea) [19].

Assuming a clinically relevant difference in mean pain intensity score of 2 points between intervention and control groups, with a standard deviation of 3.5 points (based on results from Tsai et al. [6, 7]), the sample size required for 90% power, and α 0.05 was 69 participants per group, using a two-tailed Wilcoxon-Mann–Whitney U test (90% power and α 0.05). Including 83 patients in each group allowed for 20% loss to follow-up.

For statistical analysis, *χ*^2^ test was used for comparison of binominal variables and Mann–Whitney U test for continuous variables. Evolution of NRS scores over time was compared using analysis of variance (ANOVA) for repeated measures. Univariate analysis of variance was used to control for possible confounding factors. Results are presented as median (inter quartile range) or numbers with percentages. A *P* value<0.050 was considered statistically significant. Statistical analyses were carried out with SPSS 26.0 (SPSS Inc., Chicago, IL), except Number Needed to Treat (NNT) which was calculated with QuickCalcs (GraphPad Software, San Diego, CA).

## Results

**Fig. 1:**
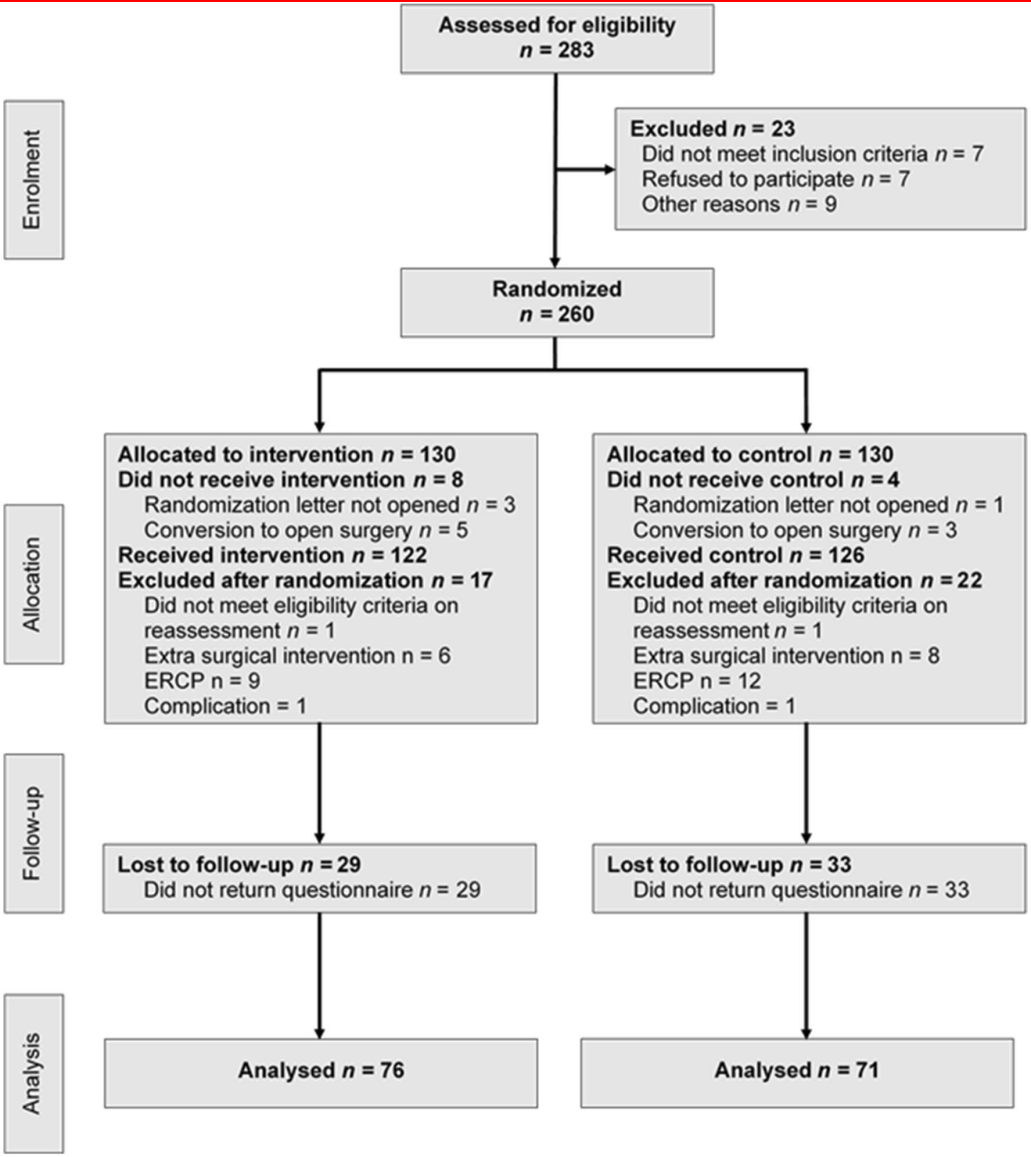
CONSORT flow chart

Participants were recruited as of December 2014 until December 2018. Loss to follow-up, due to exclusion or missing questionnaires, was greater than expected. The Regional Ethical Review Board granted our request to randomize another 47 patients to each group (2018/2-32). Consequently, 260 participants were included and randomized, 130 to PRM and 130 to control (Fig. 1).

Of 39 excluded participants, 21 had an ERCP, 14 had an extra surgical intervention (5 closures of mesenteric defects after previous bariatric surgery, 2 umbilical hernia repairs, 3 had extra ports because of intra-abdominal adhesions obstructing the view, and 4 received tube drainage (3 because of subtotal cholecystectomy)). Two had a complication; both postoperative infections: one requiring IV antibiotics and percutaneous drainage (CD III), and one with concomitant pancreatitis (CD II). Two participants should not have been enrolled, due to acute cholecystitis and pancreatitis, respectively.

Finally, 147 questionnaires were available for analysis, 76 in the intervention group (64 completed, 12 partially completed), and 71 in the control group (63 completed, 8 partially completed). A non-response analysis was performed on all available data, see Appendix. Apart for a shorter duration of surgery in the “loss to follow-up” group (85.5 (69.0–109.3) minutes compared to 95.0 (81.0–114.0) minutes), the analysis did not show any significant differences between the “loss to follow-up” group and the group with questionnaires.

Baseline characteristics were similar between the groups (Table 1). Clinical data are presented in Table 2. The only parameter with a significant difference between the groups was a somewhat longer duration of surgery in the PRM group. No pulmonary complication was observed.

**Table 1:** Patient characteristics at baseline

	PRM	Control
Age (years)	44.5 (36.3–58.8)	46.0 (37.0–57.0)
BMI (kg/m^2^)	27.2 (24.6–31.8)	27.4 (24.0–30.9)
Gender ratio (female: male)	56 (73.7%): 20 (26.3%)	56 (78.9%): 15 (21.1%)
Indication for surgery		
-Previous cholecystitis	5 (6.6%)	3 (4.2%)
-Previous pancreatitis	2 (2.6%)	4 (5.6%)
-Gallstone attacks only	70 (92.0%)	65 (91.2%)
Previous abdominal surgery	32 (42.1%)	28 (39.4%)
Fibromyalgia	4 (5.3%)	1 (1.4%)
Chronic pain	19 (25.0%)	18 (25.4%)
Regular analgesic consumption	10 (13.2%)	8 (11.3%)
-Paracetamol	7 (9.2%)	7 (9.9%)
-NSAID	4 (5.3%)	4 (5.6%)
-Opioid	1 (1.3%)	2 (2.8%)

Data are expressed as median (interquartile range) or number (percentage)

PRM = Pulmonary recruitment maneuver; BMI = Body mass index; NSAID = Nonsteroidal anti-inflammatory drug

**Table 2:** Surgical characteristics

	PRM	Control	P*
Surgeon attending: resident	42 (55.3%): 34 (44.7%)	45 (63%): 26 (37%)	0.317
Anesthesia maintenance analgesic remifentanil: fentanyl	75 (98.7%): 1 (1.3%)	69 (97.2%): 2 (2.8%)	0.520
Anesthesia maintenance hypnotic propofol: sevoflurane	23 (30.3%): 53 (69.7%)	13 (18.3%): 58 (81.7%)	0.092
Duration of surgery (minutes)	98 (82.8–120.0)	93 (78.0–106.0)	0.027
Estimated blood loss (mL)	0 (0–5)	0 (0–0)	0.593
Ondansetron 4 mg preoperative	31 (40.8%)	21 (29.6%)	0.155
Ketorolac 30 mg	61 (80.3%)	55 (77.5%)	0.678
Morphine	74 (97.4%)	71 (100%)	0.169
Morphine dose (mg/kg)	0.1 (0.1–0.1)	0.1 (0.1–0.1)	0.131
Local anesthetic	76 (100%)	71 (100%)	0.999

Data are expressed as median (interquartile range) or number (percentage)

PRM = pulmonary recruitment maneuver

*χ^2^ test for binominal variables; Mann–Whitney U test for continuous variables

**Fig. 2:**
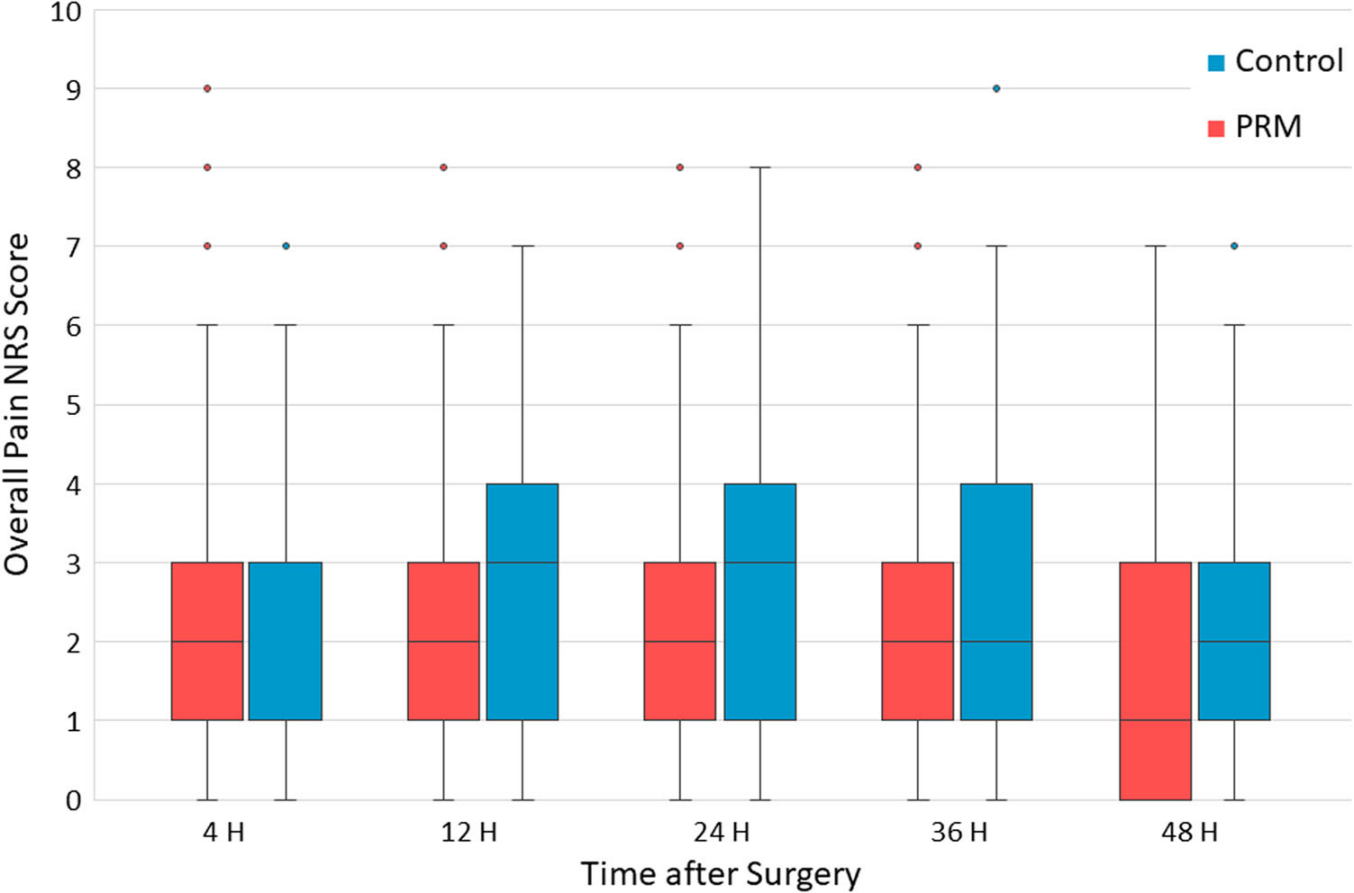
Overall pain intensity

Incidence of overall pain did not differ significantly between the groups on any occasion. For the postoperative 48-h period, the incidence of overall pain (NRS 1–10) was 98.7% in the PRM group and 94.4% in the control group (*P*=0.149). Overall pain intensity did not differ over time (*P*=0.739), nor at a certain hour (Fig. 2).

**Fig. 3:**
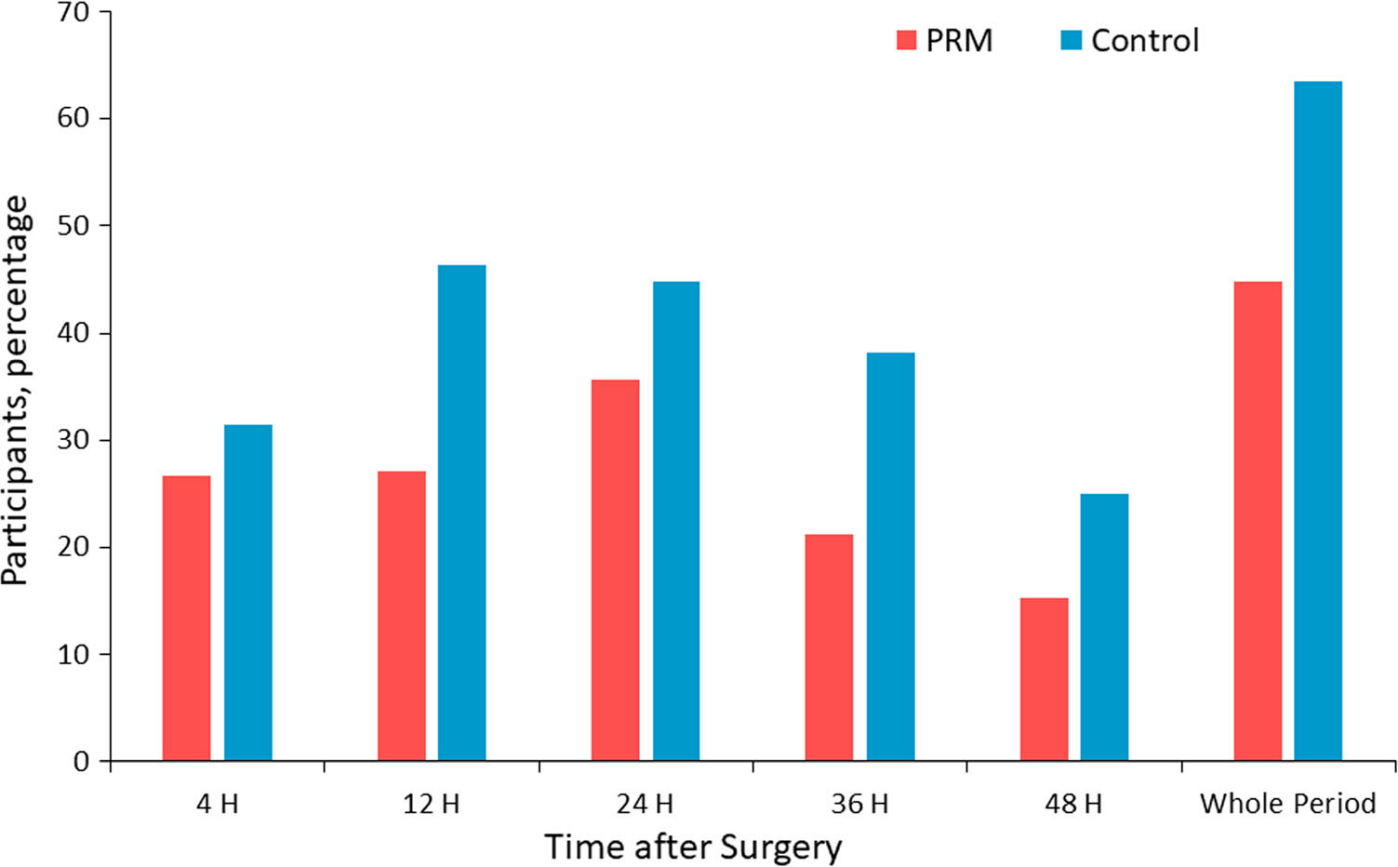
Shoulder pain incidence

There was a significant difference in shoulder pain incidence favorable for the PRM group. During the 48 h after surgery, 44.7% suffered from shoulder pain in the PRM group, versus 63.4% in the control group (*P*=0.023). The Number Needed to Treat, NNT, to benefit from PRM was 6 (95% Confidence Interval, CI, 2.9–35.5). The incidence in shoulder pain was specifically lower in the PRM group 12 and 36 h after surgery (PRM 27.0% vs. control 46.4% at 12 h [*P*=0.016] and PRM 21.1% vs. control 38.2% at 36 h [*P*=0.027]) (Fig. 3). NNT was 6 (95% CI 2.9–26.1) at 12 h and 6 (95% CI 3.1–46.4) at 36 h after surgery.

Incidence of wound pain differed at 4 and 24 h postoperatively, in favor of the control group (25.7% vs. 41.3% [*P*=0.047] and 41.8% vs. 60.3% [*P*=0.029]). The difference was not significant when analyzing the occurrence of wound pain during the whole 48-h period after surgery; 67.1% experienced wound pain in the PRM group, versus 54.9% in the control group (*P*=0.130).

Incidence of nausea (NRS 1–10) during the 48-h period after surgery was 51.3% in the PRM group and 70.4% in the control group (*P*=0.018). The incidence of nausea was specifically lower in the PRM group 4 and 12 h after surgery (PRM 33.8% vs. control 55.7% at 4 h [*P*=0.011] and PRM 24.7% vs. control 43.5% at 12 h [*P*=0.018]). The NNT to benefit from reduced nausea incidence was 6 (95% CI 2.9–27.4) during the 48-h period, 5 (95% CI 2.6–16.5) 4 h after surgery, and 6 (95% CI 2.9–28.5) 12 h after surgery.

**Fig. 4:**
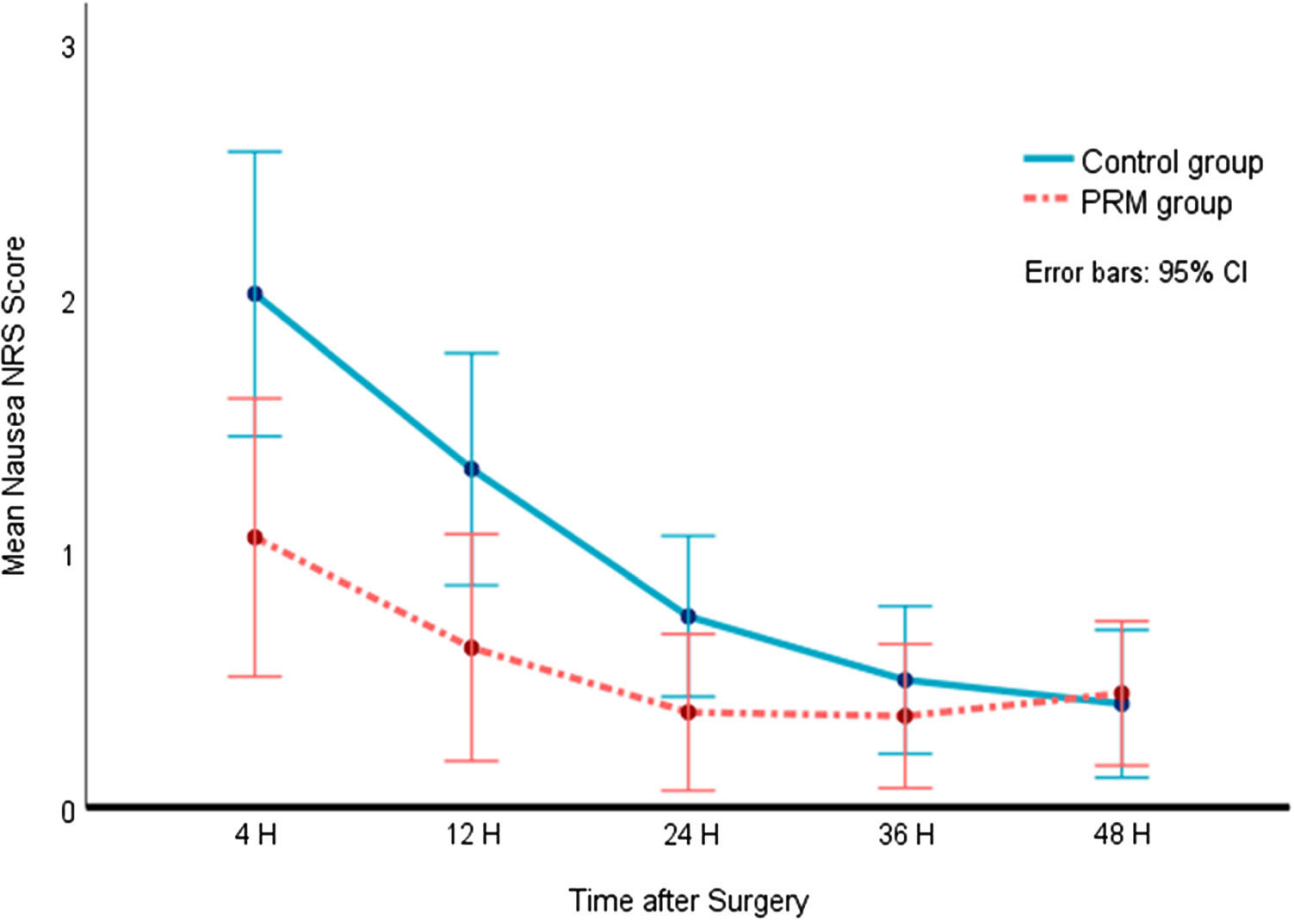
Nausea intensity

Nausea intensity was low in both groups, though differed significantly between them during the 48 postoperative hours (*P*=0.025) (Fig. 4). At 4 h, after surgery NRS values were 0 (0.0–2.0) in the PRM group and 1 (0.0–4.0) in the control group (*P*=0.006), and at 12 h, 0 (0.0–0.5) in the PRM group and 0 (0.0–2.5) in the control group (*P*=0.015).

No one in the PRM group had vomited 4 h after surgery, while 13.0% had vomited in the control group (*P*=0.001). At 12 h, 9.6% had vomited in the PRM group and 16.4% in the control group (*P*=0.228). Vomiting later was rare. At 4 h, after surgery 94.5% of the participants in the PRM group were up on their feet, while only 82.6% in the control group (*P*=0.025).

Opioid consumption during hospital stay postoperatively did not differ significantly (Table 3).

**Table 3:** Postoperative characteristics

	PRM	Control	P*
Opioid, IV and, or tablet	51 (67.1%)	40 (56.3%)	0.179
Opioid IV	43 (56.6%)	30 (42.3%)	0.083
Opioid IV, total mg	2.0 (0.0–4.0)	0.0 (0.0–3.0)	0.103
Oxycodone tablet	33 (43.4%)	30 (42.3%)	0.886
Oxycodone tablet, total mg	0 (0.0–5.0)	0 (0.0–5.0)	0.969
NSAID per os	1 (1.3%)	7 (9.9%)	0.023
Ondansetron	19 (25.0%)	30 (42.3%)	0.027
Ondansetron, mg	0 (0.0–1.5)	0 (0.0–4.0)	0.015
Hospital stay, days	1 (1–1)	1 (1–1)	0.507
Analgesic 36 h	63/71 (88.7%)	63/68 (92.6%)	0.428
Paracetamol 36 h	61/71 (85.9%)	60/67 (89.6%)	0.516
Oxycodone 36 h	18/71 (25.4%)	15/67 (22.4%)	0.683
NSAID 36 h	6/71 (8.5%)	8/67 (11.9%)	0.497
Analgesic 48 h	56/72 (77.8%)	53/65 (81.5%)	0.586
Paracetamol 48 h	55/72 (76.4%)	50/65 (76.9%)	0.941
Oxycodone 48 h	12/72 (16.7%)	8/65 (12.3%)	0.471
NSAID 48 h	5/72 (6.9%)	10/65 (15.4%)	0.114

Data are expressed as median (interquartile range) or number (percentage)

PRM = Pulmonary recruitment maneuver

*χ^2^ test for binominal variables; Mann–Whitney U-test for continuous variables

The percentage of patients receiving IV opioids postoperatively was somewhat larger in the intervention group. Although not part of the study protocol, nonsteroidal anti-inflammatory drugs (NSAID) were sometimes administered. In the PRM group 1.3% of the participants received NSAID, versus 9.9% in the control group (*P*=0.023). After discharge from hospital, there was no difference in analgesic consumption between the groups.

Antiemetics were more frequently needed in the control group postoperatively (*P*=0.027). The dose required was also higher in the control group (*P*=0.015). When adjusted for ondansetron given preoperatively, the difference between the groups was reinforced (*P*=0.003).

## Discussion

This trial is the first that evaluated the effect of PRM on postoperative pain solely in patients treated with laparoscopic cholecystectomy, and the second to use the ventilator to perform PRM [3]. We found that a PRM significantly reduced the incidence of postoperative shoulder pain during the postoperative 48-h period. This is consistent with previous studies [4–7, 9, 15]. The NNT, to benefit from reduced shoulder pain incidence was 6, similar to NNT reported by Tsai et al. [7].

In studies investigating shoulder pain after laparoscopic cholecystectomy, the prevalence has been 36–80% (control populations) [20–24]. In this study, 64.3% of the control population suffered from shoulder pain, 44.7% in the PRM group. There seems to be a positive correlation between volume of sub-diaphragmatic gas and intensity in shoulder pain [11–15, 25]. Recent studies found that PRM efficiently evacuate sub-diaphragmatic gas [13–15, 25].

Incidence of wound pain differed at two occasions, 4 and 24 h after surgery, in favor of the control group. Wound pain incidence did not differ between the groups when evaluating the entire 48-h postoperative period. Kiyak et al. found wound pain score being lower in the control group 6 h after surgery, and Davari-Tanha et al. reported lower incision site pain in the control group 24 h after surgery [25, 26]. Two studies by Tsai et al. and one study by Güngördük did not find any difference between groups regarding neither incidence nor intensity of wound pain [6, 7, 27]. The varying results, and lack of a connecting theory, leave us in doubt of a correlation. Ryu et al. evaluated the effect of PRM combined with saline instillation on postoperative pain in wounds and shoulders. Their results showed that the relative pain severity was differently perceived postoperatively. Wound pain was considered more intense than shoulder pain in the intervention group, while the control group perceived shoulder pain more intense than wound pain [14]. Possibly, not experiencing shoulder pain shifts the attention to other pain areas.

Postoperative pain was well controlled in both groups, the median NRS score being maximum 2 in the PRM group and 3 in the control group. No significant difference was found between the groups regarding intensity of overall pain, though there was a trend in favor of the PRM group. Two previous studies have analyzed overall pain, both reporting less pain in the PRM group [3, 8]. Our study design, with many members of the hospital staff involved, intending to detect differences of generalizable clinical relevance, means minor differences in the care might appear. Potentially, this could obscure the results and explain why no significant difference in overall pain was detected.

Incidence of PONV after laparoscopic cholecystectomy ranges from 53 to 72% [28]. Despite routine PONV prophylaxis, PONV was frequent, affecting 70.4% in the control group, compared to 51.3% in the PRM group. The reduction in the PRM group was significant, and the NNT to benefit from this reduction was 6 patients overall. The two previous studies analyzing PRM in patients undergoing laparoscopic cholecystectomy (among others) did not comment on postoperative nausea [8, 15]. Nausea intensity was generally low, though significantly lower in the PRM group. The PRM population was up on their feet earlier, and the requirement of antiemetics was lower, further supporting a lower intensity of nausea in this group.

Duration of surgery in the PRM group was a few minutes longer than in the control group. 10 of 11 prior studies indicate that PRM do not prolong surgery [3, 4, 6, 7, 9, 13, 14, 25–27]. A reason surgical duration was slightly longer in our PRM group could be that the operating team had varying experience of the maneuvre.

That participants were excluded after randomization, due to for example ERCPs, could have entailed imbalances in baseline characteristics. Though the participants appear similar, we do recognize this as a limitation in the study protocol.

The anesthetic management was not completely standardized. It did not differ significantly between the groups though. Induction analgesia was achieved using remifentanil or alfentanil, both having rapid onset of action and short terminal half-lives [29]. Alfentanils duration of activity is < 10–24 min after a single dose [29, 30]. Remifentanil, also used to maintain anesthesia, has a terminal elimination half-life of 6–12 min, independent of renal and hepatic function [29]. Nausea and vomiting are common adverse effects of the selected analgesic and hypnotic drugs. Minor differences in anesthetic treatment ought therefore not to have biased our results.

## Conclusions

Our study indicates that a one-minute, ventilator-piloted PRM reduces incidence of shoulder pain after laparoscopic cholecystectomy. It further suggests that the PRM result in reduced incidence and intensity of postoperative nausea, and decreased need for antiemetics. The maneuvre is safe and uncomplicated to perform.

## Supplementary Information

Below is the link to the electronic supplementary material.

Supplementary file1 (DOCX 20 kb)
