# Supplementary material for: Pulmonary Recruitment Maneuver Reduces Shoulder Pain and Nausea After Laparoscopic Cholecystectomy: A Randomized Controlled Trial
Source: World J Surg. 2021 Sep 5;45(12):3575–83. doi: 10.1007/s00268-021-06262-6 (PMC8572840; doi:10.1007/s00268-021-06262-6)
Supplement: Supplementary file 1 — Supplementary file1 (DOCX 20 kb) [file 268_2021_6262_MOESM1_ESM.docx]

| **Non-response analysis.** | | | |
| --- | --- | --- | --- |
|  | **Included** | **Loss to follow-up** | ***P**** |
| Age (years) | 46.0 (37.0-58.0) | 48.0 (34.0-58.0) | 0.935 |
| BMI (kg/m^2­^) | 27.4 (24.2-31.2) | 27.6 (25.3-31.5) | 0.470 |
| Gender ratio (female:male) | 112 (76.2%) : 35 (23.8%) | 45 (72.6%) : 17 (27.4%) | 0.581 |
| Indication for surgery |  |  |  |
| -previous cholecystitis | 8 (5.4%) | 6 (9.7%) | 0.263 |
| -previous pancreatitis | 6 (4.1%) | 3 (4.8%) | 0.805 |
| -gallstone attacks only | 135 (91.8%) | 54 (87.1%) | 0.287 |
| Previous abdominal surgery | 60 (40.8%) | 29 (46.8%) | 0.426 |
| Fibromyalgia | 5 (3.4%) | 5 (8.1%) | 0.149 |
| Chronic pain | 37 (25.2%) | 12 (19.4%) | 0.365 |
| Regular analgesic consumption | 18 (12.2%) | 13 (21.0%) | 0.105 |
| -paracetamol | 14 (9.5%) | 10 (16.1%) | 0.158 |
| -NSAID | 8 (5.4%) | 6 (9.7%) | 0.250 |
| -opioid | 3 (2.0%) | 2 (3.2%) | 0.596 |
| Surgeon  attending : resident | 87 (59.2%) : 60 (40.8%) | 43 (69.4%) : 19 (30.6%) | 0.166 |
| Anesthesia  maintenance analgesic  remifentanil : fentanyl | 144 (98.0%) : 3 (2.0%) | 60 (96.8%) : 2 (3.2%) | 0.609 |
| Anesthesia  maintenance hypnotic  propofol : sevoflurane | 36 (24.5%) : 111 (75.5%) | 21 (33.9%) : 41 (66.1%) | 0.164 |
| Duration of surgery (minutes) | 95.0 (81.0-114.0) | 85.5 (69.0-109.3) | 0.032 |
| Estimated blood loss (mL) | 0.0 (0.0-5.0) | 0.0 (0.0-20.0) | 0.058 |
| Ondansetron 4 mg preoperative | 52 (35.4%) | 27 (43.5%) | 0.266 |
| Ketorolac 30 mg | 116 (78.9%) | 45 (72.6%) | 0.320 |
| Morphine | 145 (98.6%) | 62 (100%) | 0.356 |
| Morphine dose (mg/kg) | 0.1 (0.1-0.1) | 0.1 (0.1-0.1) | 0.726 |
| Local anesthetic | 147 (100%) | 62 (100%) | 0.999 |
| Opioid, IV and, or tablet | 91 (61.9%) | 35 (56.5%) | 0.462 |
| Opioid IV | 73 (49.7%) | 31 (50.0%) | 0.964 |
| Opioid IV, total mg | 0.0 (0.0-3.0) | 1.0 (0.0-5.0) | 0.603 |
| Oxycodone tablet | 63 (42.9%) | 23 (37.1%) | 0.440 |
| Oxycodone tablet, total mg | 0.0 (0.0-5.0) | 0.0 (0.0-6.3) | 0.892 |
| NSAID per os | 8 (5.4%) | 4 (6.5%) | 0.774 |
| Ondansetron | 49 (33.3%) | 20 (32.3%) | 0.880 |
| Ondansetron, mg | 0.0 (0.0-4.0) | 0.0 (0.0-4.0) | 0.987 |
| Hospital stay, days | 1.0 (1.0-1.0) | 1.0 (1.0-1.0) | 0.670 |

Data are expressed as median (interquartile range) or number (percentage).

* *χ*^2^ test for binominal variables; Mann–Whitney U-test for continuous variables.
